# Knockout of the S-acyltransferase Gene, *PbPAT14*, Confers the Dwarf Yellowing Phenotype in First Generation Pear by ABA Accumulation

**DOI:** 10.3390/ijms20246347

**Published:** 2019-12-16

**Authors:** Hongguang Pang, Qi Yan, Shuliang Zhao, Fang He, Jianfeng Xu, Baoxiu Qi, Yuxing Zhang

**Affiliations:** 1 Pear Engineering and Technology Research Center of Hebei, College of Horticulture, Hebei Agricultural University, Baoding 071001, China; pangpear@163.com (H.P.);; 2 College of Biological Sciences and Technology, Beijing Forestry University, Beijing 100083, China

**Keywords:** ABA accumulation, CRISPR/Cas9, dwarf yellowing phenotype, pear breeding, protein S-acyltransferases

## Abstract

The development of dwarf fruit trees with smaller and compact characteristics leads to significantly increased fruit production, which is a major objective of pear (*Pyrus bretschneideri*) breeding. We identified the S-acylation activity of *PbPAT14*, an S-acyltransferase gene related to plant development, using a yeast (*Saccharomyces cerevisiae*) complementation assay, and also *PbPAT14* could rescue the growth defect of the *Arabidopsis* mutant *atpat14*. We further studied the function of *PbPAT14* by designing three guide RNAs for *PbPAT14* to use in the CRISPR/Cas9 system. We obtained 22 positive transgenic pear lines via *Agrobacterium*-mediated transformation using cotyledons from seeds of *Pyrus betulifolia* (‘Duli’). Six of these lines exhibited the dwarf yellowing phenotype and were homozygous mutations according to sequencing analysis. Ultrastructure analysis suggested that this dwarfism was manifested by shorter, thinner stems due to a reduction in cell number. A higher level of endogenous abscisic acid (ABA) and a higher transcript level of the ABA pathway genes in the mutant lines revealed that the *PbPAT14* function was related to the ABA pathway. Overall, our experimental results increase the understanding of how PATs function in plants and help elucidate the mechanism of plant dwarfism.

## 1. Introduction

The current trend in global pear fruit production is to cultivate grafted trees in modern orchards by grafting pear cultivar scions onto dwarfing rootstock [1]. The growth and development of cultivar scions are limited by the dwarfing rootstocks to reduce tree size and thus densely plant trees in orchards [2,3]. As dwarfing rootstocks simplify orchard management and reduce production costs, there is great interest in producing elite dwarfing rootstocks for pear and understanding the basic mechanism of plant dwarfism [4,5]. Many attempts have been made to exploit dwarfing rootstocks in pear, but the research progress to date has been stagnant due to scion incompatibility, reduced abiotic stress tolerance, and heightened susceptibility to disease [6,7,8,9]. In particular, an effective dwarfing rootstock for Asian pear cultivars has yet to be established [10]. Because dwarfism is a complex trait controlled and influenced by multiple genes, there are currently many difficulties in creating dwarfing rootstocks using traditional crossing and breeding techniques [2]. Moreover, pear dwarfing rootstock germplasm resources are limited [5].

Several genes associated with the dwarfing effect have been molecularly analyzed to understand the regulation of dwarfism in various plant species. For example, in *Arabidopsis*, the mutant *sax1*, loss-of-function *SAX1*, is associated with dwarfism, delayed development, and reduced fertility [11]. Additionally, the mutant *acl5* causes severe dwarfism and inhibits stem elongation [12]. For apple, *Dw1*, *Dw2*, and *Dw3* are associated with rootstock-induced dwarfing by quantitative trait locus (QTL) analyses, but these genes have not been investigated for dwarfing functions [13,14]. Several genes have been identified as dwarfing genes in pear using RNA-seq analysis, such as *GA3ox*, *ZEP*, *ERF073*, etc., but these results remain inconsistent, particularly in regards to the analysis of different pear cultivars using the qRT-PCR method [5].

In addition to genes, it has been reported that plant hormones play a role in the regulation of dwarfism including, indole-3-butyric acid (IBA), indole acetic acid (IAA), benzyladenine (BA), abscisic acid (ABA), gibberellin (GA), and brassinosteroid (BR) [15,16,17]. Usually, the dwarfing phenotype is a result of reduced cell division and/or cell elongation [18]. *Arabidopsis* mutants deficient in GA biosynthesis, such as *ga1*, *ga4*, and *ga5*, markedly show the dwarf phenotype with short roots and delayed flowering [19,20]. Overexpression of *MdWRKY9* leads to dwarfing in apple rootstock M26 (*Malus pumila*), along with lower transcription levels of *MdDWF4* and lower levels of BR [16]. In addition, the ABA concentration of bark in dwarf apple and citrus is higher than that of taller varieties, and treatment with exogenous ABA results in shortened internodes and decreased growth in the two apple species (*Malus sieversii* and *Malus hupehensis*) [21,22]. S-acylation is a post-translational lipid modification process occurring in eukaryotes that regulates trafficking, regulation, signaling, membrane association, and target protein functions [23]. S-acylation is catalyzed by a family of enzymes called Protein S-acyltransferases (PATs) [24,25]. PATs are transmembrane proteins and contain DHHC-CRD domains with a broad expression pattern in different developmental stages and tissues in all analyzed plants [24,26,27]. Previous investigations demonstrated that PATs could regulate plant growth, development, and stress responses via different signaling paths, such as Ca^2+^ signaling, hormone signaling, intracellular trafficking, etc. For example, in *Arabidopsis*, *AtPAT10*-mediated S-acylation is critical for cell expansion, fertility, and salt stress through the actions of calcineurin B-like proteins (CBLs) [28,29]. Additionally, *AtPAT24* (*Tip1*) loss-of-function results in defective growth of pollen tubes and root hairs [30]. Moreover, *AtPAT14* functions are involved in stem and leaf development via endogenous hormone signaling [31]. *AtPAT15* functions are related to β-oxidation of seed storage triacylglycerol during early seedling growth [32]. *AtPAT21*, another PAT reported recently, participates in both male and female gametogenesis [33]. Further, overexpression of *OsPAT15* results in increased branch and seed yield in *Brassica napus* L. [34]. However, our understanding of plant S-acylation remains limited due to a large number of PATs and an even larger number of putative S-acylated substrate proteins in plants.

To date, as a highly efficient and powerful genome modification tool for breeding programs, the clustered regularly interspaced short palindromic repeats-associated systems (CRISPR/Cas9) has been widely utilized to edit the genomes of various major crops. For instance, the *clv3* tomato mutant generated by the CRISPR/Cas9 system produced more organs and larger fruits than wild-type tomato plants [35]. Moreover, knockout of *EIF4E* increased cucumber immunity to multiple viruses, including cucumber vein yellowing virus, zucchini yellow mosaic virus, and papaya ringspot mosaic virus [36]. CRISPR/Cas9-mediated gene editing of *VvWRKY52* in grape increased its resistance to *Botrytis cinerea* infection [37]. Despite these successes, it remains a challenge to produce homozygous mutations in woody plants with long reproductive cycles in the first generation, which are especially important for successful genetic breeding using this system [37]. Consequently, to date, the only report of pear gene editing via CRISPR/Cas9 focused on the *PcTFL1.1* gene using apple *TFL1.1* gRNAs, indicating nonetheless that the CRISPR/Cas9 mediated knockout of targeted genes is possible in pear [38].

In this present work, our aims were to: (i) Determine whether homozygous mutant lines in pear could be efficiently generated using CRISPR/Cas9 technology, (ii) observe the phenotype of *PbPAT14* knockout mutant *pbpat14*, and (iii) explore the signal pathway modified by PbPAT14 in pear. For these purposes, we searched the homologous *AtPAT14* gene in pear using local BLASTP software and further identified its S-acylation activity using yeast and *Arabidopsis* complementation assays. Three different single guide RNAs (sgRNAs) were designed and associated with the Cas9 nuclease for *PbPAT14*. We obtained transgenic plants from cotyledon callus cells from the seeds of a widespread pear species (*Pyrus betulifolia*, ‘Duli’) via *Agrobacterium*-mediated transformation. Analysis of the first-generation transgenic plants verified 6 homozygous mutant lines of the 22 transgenic plants, indicating that the generation of pear homozygous mutant lines in the first generation via the CRISPR/Cas9 system is feasible. Furthermore, the endogenous hormone test and gene expression analysis showed that *PbPAT14* functions modified the ABA pathway. S-acylated proteins were further identified from poplar using a proteomics method and CPKs were thus further designated as *PbPAT14* putative substrate modified proteins.

## 2. Results

### 2.1. Identification and Molecular Characterization of the PbPAT14 Gene in Pear

**Figure 1:**
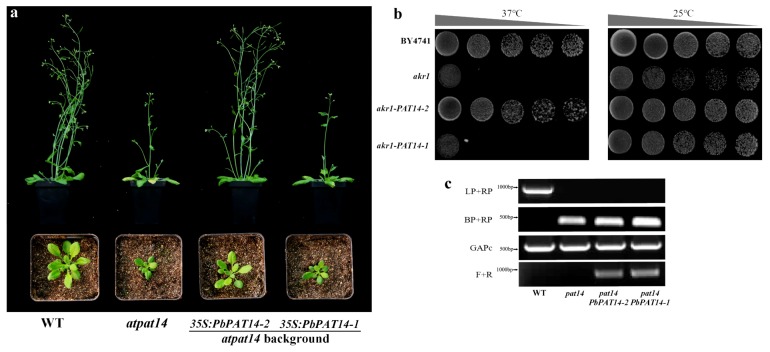
The phenotype of transgenic *Arabidopsis* mutant *atpat14* and yeast mutant *akr1*. (**a**) PbPAT14-2 can rescue the growth defect of *Arabidopsis* mutant *atpat14* (SALK_026159), but PbPAT14-1 cannot do so. Six- (top) and three-week-old (bottom) wild-type *Columbia-0 (Col-0)*, mutant *Arabidopsis* (*atpat14*), and transgenic *Arabidopsis* mutant plants (*pat14-PbPAT14-1* and *pat14-PbPAT14-2*). (**b**) PbPAT14-2 can rescue growth defects of the temperature-sensitive yeast mutant *akr1* that lacks the DHHC-PAT AKR1, but PbPAT14-1 cannot do so. The wild-type yeast BY4741 and *akr1* to act as the positive and negative controls. The grey triangles represent a decrease in yeast concentration from left to right. (**c**) Amplification of the T-DNA insert region of the *AtPAT14* transcript in wild-type *Col-0* and mutants (*atpat14*, *pat14-PbPAT14-1*, *pat14-PbPAT14-2*). *GAPc* (AT3G04120) served as a control. The primer pairs are shown in the left column. F/R represents PbPAT14-1F/R and PbPAT14-2F/R for *pat14-PbPAT14-1* and *pat14-PbPAT14-2*, respectively.

The phylogenetic analysis and analysis of multiple alignments revealed that two candidate *PbPAT14* proteins (PbPAT14-1 and PbPAT14-2) and AtPAT14 were clustered with a high bootstrap value (Figure S1). Moreover, these shared the DHHC-CRD domain sequence, C-X2-C-X4-P-X1-R-X2-HC-X2-C-X2-C-X4-DHHC-X1-W-X3-C-X1-G-X2-NY-X2-F, suggesting their evolutionary conservation (Figure S2a). Yeast complementation method has been used previously to test the activity of PATs in *Arabidopsis* and rice. In our study, yeast complementation results showed that PbPAT14-2 could rescue the growth defect of the yeast mutant *akr1* at 37 °C, whereas PbPAT14-1 cannot do so, suggesting that PbPAT14-2 can exhibit PAT activity (Figure 1b). Further, the transgenic *Arabidopsis* mutant *atpat14,* which possessed the PbPAT14-2 protein, resembled wild-type *Columbia-0 (Col-0)* (Figure 1a,c), suggesting that PbPAT14-2 is the PbPAT14 in pear (called *PbPAT14* hereafter). In addition, the *PbPAT14* open reading frame (ORF) contained 906 nucleotides encoding a protein comprising 301 amino acids. Further structural analysis indicated that this gene had 7 exons and 6 introns (Figure S2b).

### 2.2. Target Selection and Vector Construction

The *PbPAT14* gene was located on Chromosome 4, and no other copy was found in the pear reference genome database. In addition, we found no variation among the copy number at the *PbPAT14* region in the lately published *Pyrus betulifolia* (‘Duli’) genome. Since previous studies have shown that the DHHC domain was the S-acylation functional center of PATs [25], the upstream region of the DHHC domain in the *PbPAT14* gene was targeted to create mutants. Specifically, three target sites with tandem guanosine nucleotides (PbPAT14-T1, PbPAT14-T2, and PbPAT14-T3) were chosen as sgRNA complementary sites, which were located in the first, second, and third exon of the *PbPAT14* gene, respectively. Further, PCR products sequencing results of target DNA regions showed that there was no difference in the target regions of *PbPAT14* alleles from *Pyrus betulifolia* and revealing that the target sites were accurate and available for the CRISPR/Cas9 system (Figure S3a). Three *Arabidopsis* promoter sequences, AtU3d, AtU6-1, and AtU6-29, were used to drive the expression of target sequences T1, T2, and T3, respectively. The 35S promoter was used to drive the expression of the Cas9 sequence (Figure S3b).

### 2.3. Pear Transformation and Identification of Transgenic Mutant Lines

The cotyledons of *Pyrus betulifolia (*‘Duli’) seeds were selected for embryo callus induction, a process then used for pear transformation. After the seeds were sterilized by HgCl_2_ (Figure S4a), the cotyledon was transferred to the callus initiation medium (Figure S4b). Callus generation began two weeks after culturing (Figure S4c). Established calli were co-cultured with *Agrobacterium*, which contained CRISPR/Cas9 vectors, and were then transferred to pear shoot regeneration medium to induce adventitious bud transformation (Figure S4d). Adventitious bud growth was monitored for 45 days (Figure S4e). Micropropagation of the regenerated buds was then conducted on the pear growth medium (Figure S4f). A protocol outlining transgenesis in pear using the *Agrobacterium*-mediated transformation method is provided in Figure S4g.

**Figure 2:**
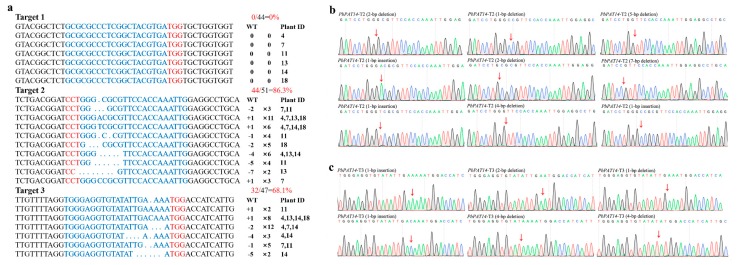
Mutation types detected in the transgenic pear lines after clustered regularly interspaced short palindromic repeats-associated systems (CRISPR)/Cas9-mediated gene editing. (**a**) A range of insertions and deletions (indels) were observed in the transgenic lines exhibiting the dwarf yellow phenotype. Blue and red sequences indicate the target sites and protospacer adjacent motif (PAM) sequences, respectively. The red number on the right represents the number of clones associated with each mutant type. The numbers of clones detected with this mutation and the plant line number are shown in black. Sequence peaks of representative mutation types at target sites for PbPAT14-T2 (**b**) and PbPAT14-T3 (**c**). Red arrows point to the locations of the mutations.

In total, 22 regenerated plants were obtained, and genomic DNA was then extracted from their leaves. PCR was performed to confirm the presence of the transgene using vector-specific primers. Results showed that all 22 putative transgenic lines (Line 1–22) tested positive for the transgene (Figure S5). Notably, six transgenic lines (Line 4, 7, 11, 13, 14, and 18) exhibited the dwarf yellowing phenotype (Figure S6). To determine whether the dwarf yellowing phenotype was caused by *PbPAT14* mutations in the transgenic lines, the putative edited area of *PbPAT14* targets were amplified by gene-specific primers from all six dwarf yellowing transgenic lines and the PCR products were then sequenced. The sequenced results of all six transgenic lines showed superimposed sequencing chromatograms, suggesting that these transgenic lines indeed contained mutations (Figure S7). To determine the types of mutations in the *PbPAT14* gene, the PCR products from each target sequence of each transgenic line were inserted into the PMD19-T Vector and 5-10 clones of each target from each line were sequenced (Table S6). According to the sequence results, various insertions or deletions (indels) existed within the *PbPAT14* mutant alleles at the desired target sites (Figure 2b,c) as a result of repair by non-homologous end joining (NHEJ) following sgRNA-directed Cas9 cleavages. We also found that the three sgRNA pairs contributed differently to *PbPAT14* mutations (Figure 2a).

### 2.4. Analysis of Potential Off-Target Mutations

The potential off-target sites of the four targets with four mismatches were predicted using a local Cas-OFFinder software (Table S4). These off-target sites were analyzed in the six transgenic lines with the dwarf yellow phenotype. However, we only amplified three off-target sequences successfully. The PCR products were purified and sequenced using the forward primers. Mutations were not detected in any of the six transgenic plant lines (Table S4, Figure S8).

### 2.5. Dwarf Yellowing Phenotype of the pbpat14 Mutant Lines

**Figure 3:**
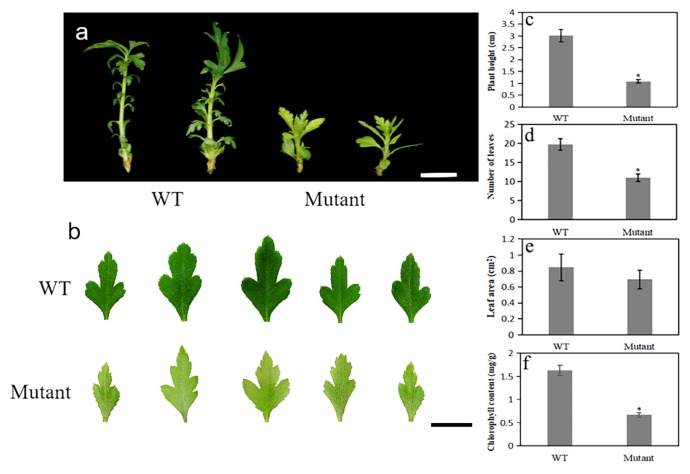
The phenotype of pear wild-type (WT) and mutant lines. The pear mutant *pbpat14* was smaller and yellower than the WT (**a**,**b**). The mutant *pbpat14* exhibited changes in plant height (**c**), leaf number (**d**), and chlorophyll content (**f**), but the leaf area showed no significant change (**e**). Scale bars, 1 cm. Data are reported as means with standard deviations (*n* = 3). The asterisks above the bars indicate a significant difference between these data (*t*-test, *p* <0.05).

Compared to the wild-type (WT), the pear mutant *pbpat14* resulted in the dwarf yellowing phenotype (Figure 3a,b). To better characterize the phenotypic differences among the mutant lines and WT, we randomly selected 3 individual 40-day-old plants of mutant (Line 4, 14, and 18) and WT plants for further analysis. On average, mutant plants were shorter (1.09 cm) than WT plants (3.01 cm) (Figure 3c). Additionally, on average, mutant plants possessed only 11 leaves, whereas WT plants possessed 19. (Figure 3d). However, there was no significant difference in the average leaf area of the leaf line-ups (5 leaves, the 1st to the 5th leaf from the apex) between the mutant lines and WT plants (Figure 3e). Chlorophyll content was further measured to quantify the yellowing phenotype. The average chlorophyll content was 0.67 mg/g in mutant lines versus 1.63 mg/g in WT plants (Figure 3f).

### 2.6. Ultrastructure Analysis of Stems and Leaves

**Figure 4:**
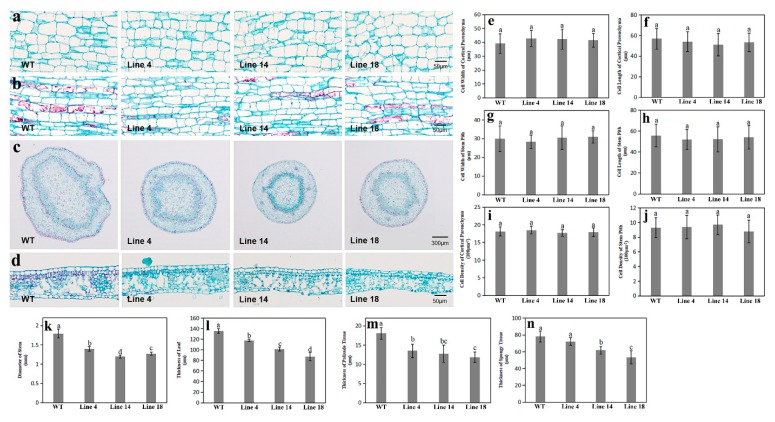
The anatomical structure of stems and leaves from pear mutant lines and WT. Longitudinal paraffin sections (**a,b**), transverse paraffin sections of stems (**c**), and transverse paraffin sections of leaves from mutant lines and WT (**d**) analyzed using light microscopy. Genotypes did not differ significantly in the longitudinal widths of both stem cortical parenchyma cells (**e**) and stem pith parenchyma cells (**g**), or in the lengths of both stem cortical parenchyma cells (**f**), and stem pith parenchyma cells (**h**). Cell density of stem cortical parenchyma cells (**i**), and pith parenchyma cells (**j**) also did not significantly differ among genotypes. However, the mutant lines possessed thinner stems (**k**), leaves (**l**), palisade tissue (**m**), and spongy tissue (**n**) when compared with WT. Data are reported as means with standard deviations (*n* = 3). Different letters above the bars indicate significant difference among these data (ANOVA, Tukey’s test, *p* < 0.05). Subset labels a, b, and c designate one or more data sets with the largest, second-largest, and third-largest mean value(s), respectively.

To observe the microstructure differences among the mutant and WT plants, leaf and stem sections of mutant and WT plants were examined and compared under a light microscope. Longitudinal stem sections revealed no significant difference in the size of cortical or stem pith parenchyma cells (Figure 4e–h) between mutant lines and WT plants (Figure 4a,b). However, the stem diameters of mutant lines were significantly thinner than those of WT plants (1.39 mm for Line 4, 1.19 mm for Line 14, 1.26 mm for Line 18, and 1.79 mm for WT) (Figure 4k). Parenchyma cell densities in the cortex and pith were further calculated, but no significant differences were found among the two genotypes (Figure 4i,j). Thus, these results suggested that the decrease in stem diameter was associated with the decline in cell numbers. In addition, we found that the leaves of mutant lines were significantly thinner than those from the WT plants (117.89 μm for Line 4, 101.54 μm for Line 14, 87.33 μm for Line 18, and 135.8 μm for WT) (Figure 4d,l). Moreover, the mutant lines possessed markedly thinner palisade and spongy tissues than the WT plants. (Figure 4m,n).

### 2.7. Knockout of PbPAT14 Induced ABA Accumulation

**Figure 5:**
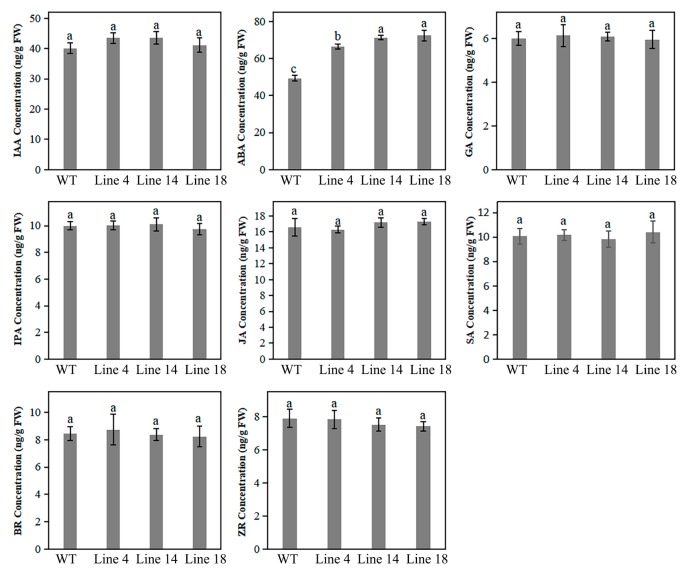
Concentrations of various endogenous hormones in mutant lines and WT, including indole acetic acid (IAA), abscisic acid (ABA), gibberellin (GA), indolepropionic acid (IPA), jasmonate (JA), salicylic acid (SA), brassinosteroid (BR), and zatin ribosie (ZR) in pear mutant lines and WT. Data are reported as means with standard deviations (*n* = 3). Different letters above the bars indicate significant difference among these data (ANOVA, Tukey’s test, *p* < 0.05). Subset labels a, b, and c designate one or more data sets with the largest, second-largest, and third-largest mean value(s), respectively.

The concentration of ABA was significantly higher in the mutant lines, whereas the levels of other plant hormones including, IAA, GA, IPA (indolepropionic acid), JA (jasmonate), SA, BR, and ZR (zeatin riboside) only slightly differed between mutant lines and WT (Figure 5). Concentrations of ABA ranged from 66 ng/g FW to 72 ng/g FW in the mutant lines versus 49 ng/g FW in the WT.

**Figure 6:**
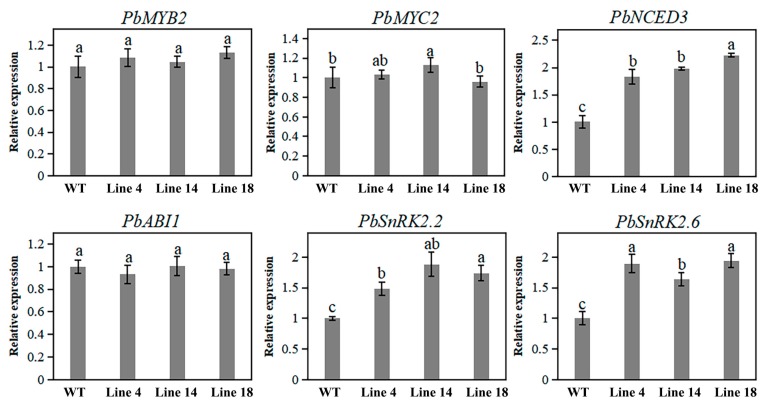
Relative expression of genes related to the ABA pathway in mutant lines versus WT determined using quantitative real-time PCR (qRT-PCR) analysis. Data are reported as means with standard deviations (*n* = 3). Different letters above the bars indicate significant difference among these data (ANOVA, Tukey’s test, *p* < 0.05). Subset labels a, b, and c designate one or more data sets with the largest, second-largest, and third-largest mean value(s), respectively.

Furthermore, genes involved in ABA pathways were selected, including *MYB2*, *MYC2*, *ABI1*, *SnRK2*, and *NCED3*. We then monitored the expression levels of their corresponding homologous genes in the mutant lines and WT using qRT-PCR. Transcript levels of *PbNCED3, PbSnRK2.2*, and *PbSnRK2.6* were significantly higher in the mutant lines than in the WT, whereas the transcript level of *PbMYC2* was only slightly higher in the mutant lines. Alternatively, the transcript levels of *PbABI1, PbMYB2,* and *PbMYC2* did not significantly differ among the mutant lines and WT (Figure 6). 

## 3. Discussion

Previous studies have indicated that PAT *AtPAT14* plays an important role in plant development via hormone signaling in *Arabidopsis* [25]. Therefore, to better understand *PbPAT14* functions in woody plants, we generated the pear PbPAT14 knockout mutant *pbpat14* using the CRISPR/Cas9 method. The pear mutant lines exhibited the dwarf yellowing phenotype compared to the WT. In addition, the mutant lines had higher levels of ABA and consistent expression of ABA pathway genes, which might explain the dwarf yellowing phenotype observed in the pear mutant lines and further suggest that *PbPAT14* functions modify the ABA pathway in pear. Although the pear mutant lines exhibited a yellowing phenotype, these findings nonetheless reveal that *PbPAT14* functions as a valuable genetic resource for modern pear production, given its role in pear dwarfism.

The putative *PbPAT14s* and *AtPAT14* are homologous given their conserved DHHC-CRD domain. *PbPAT14-1* shares 83% identity with *AtPAT14* at the amino acid level, while *PbPAT14-2* shares 70% identity. Although *PbPAT14-1* shares relatively higher homology with *AtPAT14* than *PbPAT14-2*, the yeast and *Arabidopsis* mutant complement assays showed that *PbPAT14-2* exhibits PAT activity, suggesting that changes have occurred in the *PAT14* gene during pear evolution. Similar results are also found in other species. For example, Akr2 is highly homologous to Akr1 with a typical DHHC-CRD domain in yeast, but there is no evidence to prove its PAT activity and the *akr2* mutant does not show any remarkable phenotype [39,40]. Among the 22 human DHHC proteins, 5 proteins (DHHC4, 11, 13, 19, and 22) cannot be proved their PAT activities [41]. In our study, *PbPAT14-1* and *PbPAT14-2* had different gene structure and motif distribution (Figure S2b,c), which may cause the functional difference in S-acylation activity, perhaps also explaining the loss of the PbPAT14-1 function.

Recently, CRISPR/Cas9 technology has rapidly developed and expanded, thus that it can be effectively used for molecular breeding of various plant species. Genetic editing at multiple sites using the simultaneous expression of two or more sgRNAs has been reported in *Arabidopsis*, rice, tomato, and others [42]. In our study, we obtained 22 transgenic plants through *Agrobacterium*-mediated transformation, and six (27%) of the 22 transgenic lines showed marked morphological differences. Sequence analysis revealed that the CRISPR/Cas9 expression system successfully targeted multiple sites in pear *PbPAT14*. The different efficiencies of the selected sgRNAs in directing Cas9 and mutating the target gene observed in pear in the current study were consistent with the results of previous similar studies on *Arabidopsis*, poplar, apple, and grape, indicating that the appropriate selection of sgRNA pairs is important to generate mutations effectively [43,44]. Sequence results of randomly selected clones from individual transgenic lines showed that, among the 98 clones that could cover target sites 2 and 3, 76 (78%) exhibited mutations in at least one of the two target sites. In fact, at least one *PbPAT14* target of the six transgenic lines was completely mutated with multiple types of mutations (Table S6), suggesting that these transgenic lines were likely homozygous mutants [45,46]. These results further indicate that the generation of double-stranded DNA breaks (DSB) by Cas9 likely occurred during an early stage in the T0 pear regeneration from transgenic calli, suggesting that the method described here is effective in producing homozygous mutations in first-generation pear [45].

Usually, the dwarf phenotype is caused by reductions in cell division and/or elongation. Autotetraploid apple plants generated via colchicine treatment display the dwarf phenotype with shorter cortical parenchyma cells when compared with corresponding WT plants [15]. However, *Arabidopsis* dwarf mutant *axr1* has longitudinal stem cells of the same length compared to their WT counterparts [47]. Thus, it can be difficult to determine the definite cause of the dwarf phenotype across multiple plant species. In our study, we found that the size of cells in the stem did not change after *PbPAT14* knockout in pear, but the stem diameters were significantly smaller. Therefore, we concluded that the pear mutant lines displayed the dwarf phenotype due to a reduction in cell number rather than cell size when compared with WT plants.

Phytohormones play critical roles in plant development and can thus regulate and elicit dwarfism through changes in their concentrations, transport, or signaling [48]. In this study, we found that ABA concentrations were significantly higher in the mutant lines than in WT plants. However, our investigation showed no obvious differences in IAA, GA, IPA, JA, SA, BR, and ZR concentrations. It has been frequently reported that dwarfism is attributable to an increased ABA level as ABA functions as a growth inhibitor [49,50]. It has further been reported that ABA promotes chlorophyll degradation in plants [51]; therefore, ABA accumulation could lead to yellowing in mutant plants. Moreover, it has been reported that reduced chlorophyll content may partially contribute to plant dwarfism [52]. Therefore, in the current study, it is likely that ABA accumulation, as a result of *PbPAT14* knockout in pear, played a large role in developing the dwarf yellowing phenotype observed in mutant lines.

Our investigation showed a marked difference in ABA concentration among the genotypes, which are not in accordance with the increased SA concentration in *Arabidopsis* mutant *atpat14*. Some reports have found that the signaling pathways of SA and ABA share some regulation mechanism for modulating specific physiological activities through Ca^2+^ dependent protein kinases (CPKs) [53,54,55,56,57]. Thus, we inferred from these results that PAT14 was further sub-functionalized in modifying hormone signal transduction for plant development during the process of evolution, probably via CPKs.

In conclusion, we demonstrated that producing pear mutants in the first generation using the CRISPR/Cas9 system is extremely feasible, and we further presented a useful protocol for molecular breeding in pear using this system. We also determined that knockout of *PbPAT14* confers a dwarf yellowing phenotype in pear mutant lines, which was manifested by cell number reduction and lower chlorophyll content. Furthermore, we noted that the concentration of endogenous ABA increased in the mutant lines, along with the expression of genes involved in its pathway. Based on proteomic studies of S-acylation in poplar, we further found that CPKs were most likely the substrate proteins for *PbPAT14*, which may help elucidate the underlying mechanism of S-acylation in plants and provide a novel view for dwarfing breeding of fruit trees.

## 4. Materials and Methods

### 4.1. PbPAT14 Identification and Phylogenetic Analysis

To initially identify the *PbPAT14* gene in pear, the complete pear genome (*Pyrus bretschneideri* Rehd.) was downloaded from the Pear Genome Project website [58]. The sequences of the *AtPATs* (*AtPAT1*–*AtPAT24*) were then downloaded from the *Arabidopsis* information resource (TAIR) website [24]. The *AtPAT14* protein sequence was then used as a query to perform a BLAST function against the original protein database with an *e*-value of <0.01. Subsequently, all obtained protein sequences were analyzed to verify the presence of the DHHC domain using Pfam and SMART [59]. After removing truncated and pseudo sequences, the top 10 protein sequences were named *PbPAT14-1* to *PbPAT14-10* (Table S1). Multiple alignments of these sequences were performed using MUSCLE (Version 3.6). A neighbor-joining phylogenetic tree was constructed using MEGA software (version 7.0) with the position model. Bootstrap analysis was conducted with 1000 replicates to assess the statistical support for each node. The distribution of exons and introns was determined with the online software GSDS. The conserved motifs were detected using the MEME software tool (Version 5.0.4) with the following parameters: 15 as the maximum number of motifs and a motif width between 6 and 200.

### 4.2. S-acylation Activity Analysis in Yeast and ARABIDOPSIS

The analysis of S-acylation activity in yeast and *Arabidopsis* for the putative *PbPAT14s* was carried out as described by Li [32]. The full-length coding regions of *PbPAT14s* were amplified from pear (*Pyrus betulifolia*) cDNA by overlapping PCR products with the primer pairs, 14-1F/14-1R, 14-2F/14-2R, and attB1/attB2 (Table S2), using PrimeSTAR Max DNA Polymerase (Takara, Dalian, China), and then cloned into the Gateway entry vector pDONR/Zeo (Invitrogen, Carlsbad, USA). The 2 plasmids were re-combined into pYES-DEST52 (Invitrogen, Carlsbad, USA) for expression in yeast (*Saccharomyces cerevisiae*) under the GAL1 promoter, and the 2 *PbPAT14* fragments were cloned into pEarleyGate104 for expression in *Arabidopsis* under the 35S promoter. The methods of transformation of yeast cells, *Arabidopsis* plants, and their subsequent growth conditions were also carried out as described previously by Li [32]. The empty pYES-DEST52 vector was transformed into the wild-type yeast BY4741 and *akr1* to act as the positive and negative controls. A series of 5- or 10-fold dilutions were made in sterile water from one OD600 of cells, and 8 μL of each dilution was spotted onto 2 identical galactose minimal agar medium plates to observe the growth of yeast. Images were digitally scanned with an incubation time of 48 h at 25 °C and 37 °C. PCR reaction was run for 30 cycles with 58 °C as the annealing temperature (PCR conditions were the same without any special instructions in this experiment).

### 4.3. CRISPR/Cas9 Target Site Selection and CRISPR/Cas9 Vector Construction

Three target sites were selected from the *PbPAT14* sequence for designing the sgRNAs based on their location in the target gene using the online tool ZiFiT Targeter (Version 4.2) [60], namely PbPAT-T1, PbPAT14-T2, and PbPAT14-T3. The CRISPR/Cas9 construct was generated according to the previously established method [61]. Briefly, the sgRNA expression cassettes were amplified by the sgRNA helper plasmids, PYLsgRNA-LacZ-AtU3b (Addgene plasmid #66199; http://n2t.net/addgene:66199; RRID: Addgene_66199), PYLsgRNA-AtU6-1 (Addgene plasmid #66202; http://n2t.net/addgene:66202; RRID: Addgene_66202), and PYLsgRNA-AtU6-29 (Addgene plasmid #66203; http://n2t.net/addgene:66203; RRID: Addgene_66203), using the target site containing sequence primers gRT#T1/AtU3b#T1, gRT#T2/AtU6-1#T2 and gRT#T3/AtU6-29#T3, respectively. The sgRNA expression cassettes were then cloned into the binary vector, pYLCRISPR/Cas9P35S-H (Addgene plasmid #66189; http://n2t.net/addgene:66189; RRID: Addgene_66189), at *Bsa1* site based on the golden gate cloning assembly protocol. The primers used in this experiment are listed in Table S3. The constructed plasmid was introduced into the *A. tumefaciens* strain, EHA105, using the freeze-thaw method.

### 4.4. Transformation and Growth of Pear

Pear seeds (*Pyrus betulifolia*) were sterilized by cleaning thoroughly under running tap water for 5 hours and then soaked in water for 24 h. These seeds were then treated with HgCl_2_ (0.1%) for 10 min under aseptic conditions and washed 5 times using sterile distilled water to remove traces of HgCl_2_. Then, the seed coats from each seed were removed, and the cotyledons were soaked in sterile distilled water for 1 to 2 min. After excess liquid on the seed surface was absorbed by sterilized paper, the cleaned cotyledons were transferred to callus initiation medium (1 × Murashige and Skoog medium (Solarbio, Beijing, China), 4mg/L Benzylaminopurine (Sigma, Saint Louis, USA), 0.05mg/L Indole-3-butyric acid (Sigma, Saint Louis, USA), 30g/L Sucrose, 6g/L Agar, pH 5.8). After 4 weeks of culture in the growth chamber without light, an embryo callus from the cotyledons was generated and used for pear transformation. *A. tumefaciens* strain, EHA105, containing the CRISPR/Cas9 vector, was used to transform the calli. The calli were inoculated by soaking them in an overnight-grown *Agrobacterium* suspension (OD600, 0.4–0.6) for 8 min. After inoculation, the calli were blotted dry using sterile paper and co-cultivated with *Agrobacterium* in the dark for 2 days on callus initiation medium. After co-cultivation, *Agrobacterium* was removed by flushing with sterile water, and then the callus was blotted dry and plated on callus initiation medium with 400 mg/L cefotaxime to dispose of residual *Agrobacterium*. After 1 week of culture without light, the calli were subcultured on pear shoot regeneration medium (1×Nitsch&Nitsch medium 1969 (Solarbio, Beijing, China), 3mg/L Benzylaminopurine, 0.1mg/L Naphthylacetic acid (Sigma, Saint Louis, USA), 30g/L Sucrose, 6g/L Agar, pH 5.8) with 9 mg/L hygromycin (Sigma, Saint Louis, USA) to induce adventitious bud transformation. The calli were transferred to fresh medium biweekly. Adventitious bud formation was monitored for 1 month. Micropropagation of the regenerated buds was then conducted on the plant growth medium (1 × Murashige and Skoog medium, 1.5mg/L Benzylaminopurine, 0.1mg/L Indole-3-butyric acid, 30g/L Sucrose, 6g/L Agar, PH 5.8), with the addition of 9 mg/L hygromycin. All cultures described above were maintained in the growth chamber at 25 °C.

### 4.5. Detection of Mutations

In total, we obtained 22 plantlets (Line 1–22) with hygromycin resistance. To identify the transgenic lines, all plantlets were detected by PCR analysis using the vector-specific primer pair VecF/VecR (Table S3). The transgenic lines with phenotypic differences (Line 4, 7, 11, 13, 14, and 18) were selected for the following procedures. The potential edited area of *PbPAT14* was amplified using gene-specific primer pairs F1/R1, F2/R2, and F3/R3 (Table S3) from these lines using the PrimeSTAR^®^ Max DNA Polymerase Kit (Takara, Dalian, China). PCR products were then purified and inserted into the PMD19-T Simple Vector (Takara, Dalian, China) for further analysis. Single clones were sequenced using the Sanger method and DNAMAN (version 7.0, Lynnon Biosoft, USA) was used for alignment analysis.

### 4.6. Off-Target Analysis

To detect off-target events, the potential off-target sites of the 3 targets were predicted using a local Cas-OFFinder software according to the pear genome data [62]. Specific primers OT1F/OT1R, OT2F/OT2R, OT3F/OT3R, and OT4F/OT4R (Table S3) were designed to amplify the DNA fragments with the potential off-target sites from the 6 transgenic lines with the dwarf yellow phenotype (Line 4, 7, 11, 13, 14, and 18), and the PCR products were purified and sequenced using the Sanger method.

### 4.7. Growth Parameters

Morphological indexes were first selected to characterize mutant lines and WT plants appropriately. Three individual 40-day-old plants of mutant lines (Line 4, 14, and 18) and WT plants were randomly selected for further analysis. We measured their height, leaf number, and average leaf area of the leaf line-ups (5 leaves, the 1st to the 5th leaf from the apex) to examine morphological differences among the genotypes. Plant height and leaf area were measured using ImageJ software. All the plants were maintained in the growth chamber (16 h day/8 h night, 130 μmol m^−2^s^−1^ light intensity, 60% relative humidity). Three replicates were included in each experiment. Significant differences (*p* < 0.05) among values were assessed by *t*-test.

### 4.8. Measurement of Chlorophyll Content

To estimate chlorophyll content, the method described by Katsiarimpa et al. was followed [63]. Briefly, fresh leaves (20 mg) of each genotype were homogenized in 500 μL of 96% ethanol in preweighed 1.5 mL microfuge tubes. After centrifugation, the supernatant was transferred to a fresh tube, and the OD at 470, 649, and 665 nm was recorded. The chlorophyll content was the sum of chlorophyll a (13.95 × OD665) − (6.88 × OD649) plus chlorophyll b (24.96 × OD649) − (7.32 × OD665), which was expressed as mg/g fresh weight. For each sample, we used 3 individual plants of mutant lines (Line 4, 8, and 18) and WT plants and performed three independent replicates. Significant differences (*p* < 0.05) among values were assessed by *t*-test.

### 4.9. Anatomical Structure Analysis

For histological analysis, stem (harvested from the plant morphological bottom) and leaf (harvested the 3rd leaf from the apex) sections from the mutant (Line 4, 14, and 18) and WT plants were fixed in an FAA solution (3.7% formaldehyde, 5% glacial acetic acid, and 50% ethanol) and placed under a vacuum for 1 h to remove air. The samples were then dehydrated in absolute ethanol (10 min), rinsed twice in xylene (20 min each time), and embedded in paraffin. After sectioning with a microtome, the samples were stained with safranin and fast green according to the method described by Ma et al. [64]. The paraffin sections were then examined under a light microscope (Olympus BX43, Japan). For stem analysis, the lengths and widths were measured for the cortical and pith parenchyma cells from longitudinal sections. The diameters of stems were determined from transverse sections, as well as the density of cortical parenchyma cells and pith parenchyma cells. For leaf analysis, the thicknesses of the leaf, palisade tissue, and spongy tissue from transverse sections. Three replicates were included in each experiment. Significant differences (*p* < 0.05) among values were assessed by ANOVA Tukey’s multiple comparison test.

### 4.10. Concentrations of Plant Hormones

The endogenous concentrations of IAA, ABA, GA, IPA, JA, SA, BR, and ZR were determined in the mutant lines and WT. Leaf samples were immediately frozen in liquid nitrogen and stored at −80 °C. Extraction, purification, and determination of these hormones were performed with indirect enzyme-linked immunosorbent assays (ELISAs), as described previously [65]. In brief, 0.2g leaf samples from each genotype were ground to powder and then extracted in 10 mL of extraction buffer (80% methanol (*v/v*), 1 mM butylated hydroxytoluene). The standard curves were manufactured using standard hormone samples. The ELISAs of each sample with the corresponding antibody were performed in a 96-well plate. After 0.5 h incubation at 37 °C, the cells were washed 4 times and added to the second antibodies. After incubation and washing steps, the color development was detected using the ELISA reader at an optical density of A490. The concentration of each hormone was then obtained by applying the same methodology as Weiler et al. [66]. The antibodies used for ELISA were prepared and supplied by the Engineering Research Center of Plant Growth Regulator, China Agricultural University (Beijing, China). Three replicates were included in each experiment. Significant differences (*p* <0.05) among values were assessed by ANOVA Tukey’s multiple comparison test.

### 4.11. qRT-PCR Analysis

Total RNA was isolated from pear leaf samples using the RNAprep Pure Plant Plus Kit (Tiangen, Beijing, China). Reverse-transcription was performed with the PrimeScript^®^ Reverse Transcriptase Kit (Takara, Dalian, China). The homologous pear genes involved in the ABA pathway were identified using the protein sequences of *Arabidopsis*. Detailed information regarding these genes is shown in Table S5. All specific primers were designed according to the coding sequences provided on the NCBI website (Table S2) and further confirmed by the corresponding melting curves with a single sharp. A pear actin gene (GenBank accession number: KT943411) was used as a standard control. The qRT-PCR analysis was performed with the detection system (Mastercycler ep realplex4, Eppendorf AG, Hamburg, Germany) using the SYBR^®^ Premix Ex Taq (Takara, Dalian, China) according to the manufacturer’s instructions. The qRT-PCR procedure was performed as follows: 30  s template predenaturation at 95  °C, 15 s template denaturation at 95  °C, 15 s primer annealing at 60  °C, and 30 s primer extension at 72  °C for 40 cycles, followed by the melting curve analysis. The normalized expression level of each gene was calculated using the 2^−ΔΔ*C*T^ method [67]. Three replicates were included in each experiment. The mean of WT independent replicates was arbitrarily set as 1, and the individual WT replicate levels relative to the mean are used to calculate the standard deviation. Significant differences (*p* < 0.05) among values were assessed by ANOVA Tukey’s multiple comparison test.

### 4.12. Statistical Analysis

Statistical Product and Service Solutions v. 17.0 (SPSS, Chicago, IL, USA) was used to analyze the experimental data. *T*-test and ANOVA Tukey’s multiple comparison tests were used to examine significant differences (*p* < 0.05) between mutant lines and WT plants. 

## Supplementary Materials

Supplementary materials can be found at https://www.mdpi.com/1422-0067/20/24/6347/s1.
