# Supplementary material for: Knockout of the S-acyltransferase Gene, PbPAT14, Confers the Dwarf Yellowing Phenotype in First Generation Pear by ABA Accumulation
Source: Int J Mol Sci. 2019 Dec 16;20(24):6347. doi: 10.3390/ijms20246347 (PMC6941133; doi:10.3390/ijms20246347)
Supplement: Supplementary file 1 [file ijms-20-06347-s001.pdf]

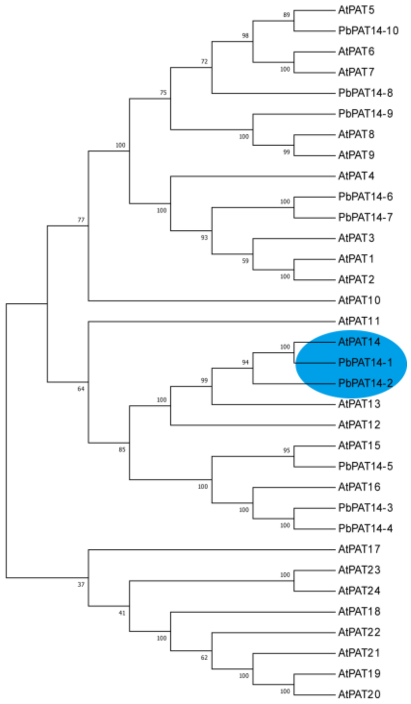

Figure S1. Phylogenetic relationships of the *Arabidopsis* AtPATs with the putative pear PbPAT14s. Bootstrap tests were performed using 1000 replicates. Horizontal branch lengths are proportional to phylogenetic distances. The candidate PbPAT14s clustered with AtPAT14 are highlighted in blue.

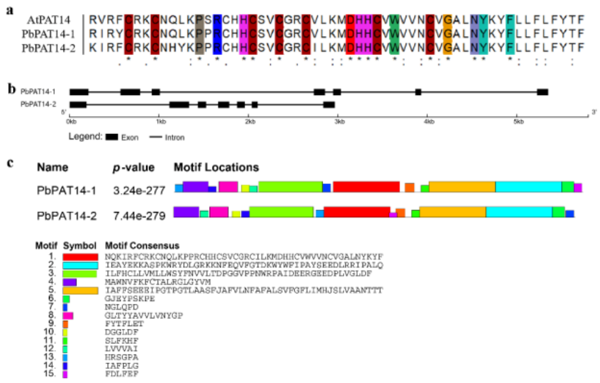

Figure S2. DHHC-CRD domain sequence, gene structure, and motif distribution from the two candidate PbPAT14s. (**a**) Alignment of the DHHC-CRD domain sequences from PbPAT14s and AtPAT14. Different colors represent different conserved amino acid residues. (**b**) Schematic representations of exon-intron compositions of *PbPAT14-1* and *PbPAT14-2*. Exons and introns are represented by black solid boxes and black lines, respectively. (**c**) Different motifs are highlighted with different colored boxes numbered 1 to 15. Lines connecting two motifs represent protein regions without detected motifs.

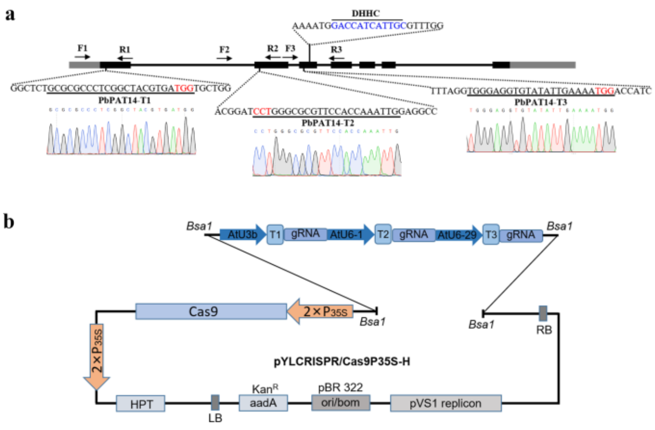

Figure S3. Target sites in the *PbPAT14* gene and schematic diagram illustrating how the Cas9/sgRNA construct was assembled. (**a**) Illustration of the three target sites (PbPAT14-T1, PbPAT14-T2, and PbPAT14-T3). Black stripe, exon. Black line, intron. Grey stripe, UTR (untranslated regions). Red and blue nucleotides represent PAM (protospacer adjacent motif) and DHHC sequences, respectively. F1/F2, R1/R2, and F3/R3 indicate primer binding sites used for PCR amplification. (**b**) Schematic diagram illustrating the construction of the three expression cassettes in the binary vector. Three *Arabidopsis* promoters (AtU3b, AtU6-1, and AtU6-29) were used to drive expression of the three target sequences (T1, T2, and T3). The three sgRNA expression cassettes were inserted into the binary vector with *Bsa1*.

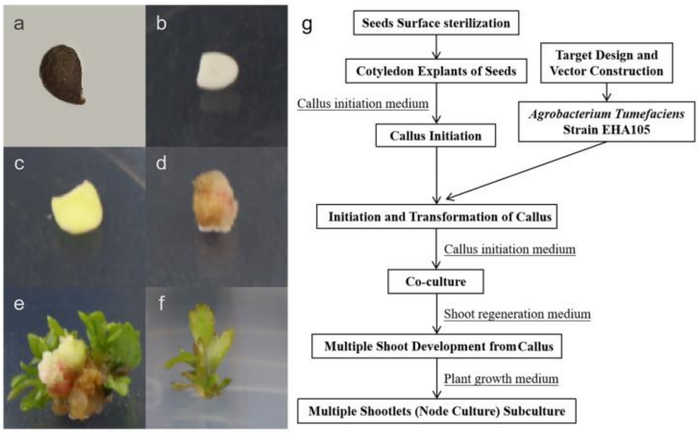

Figure S4. Callus induction and pear transformation. (**a**) Pear seeds (*Pyrus betulifolia*) were disinfected with $HgCl_2$. (**b**) Cotyledon from a pear seed. (**c**) Callus induction from cotyledons. (**d**) The well-developed calli infiltrated by the *Agrobacterium tumefaciens* strain, EHA105. (**e**) Proliferation of multiple shoots from the calli. (**f**) Plantlet formation. (**g**) A flow chart outlining the process of pear regeneration from cotyledons.

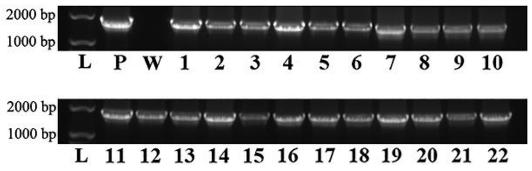

Figure S5. Identification of the 22 transgenic lines. 'P', positive control, 'W', wild-type (negative control), 'L', DNA ladder.

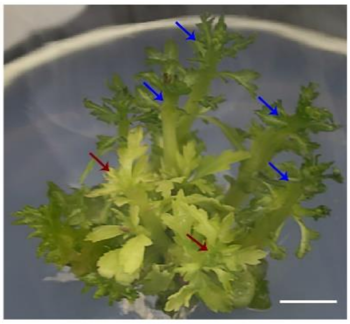

Figure S6. Phenotype of the regenerated pear shoots generated on CRISPR/Cas9-*PbPAT14* transformed calli. Red arrows indicate pear transformants with dwarf yellowing phenotype, and blue arrows indicate green shoots without this phenotype. Of the 22 transgenic lines generated, six (27%) exhibited the dwarf yellowing phenotype. Bars, 1cm.

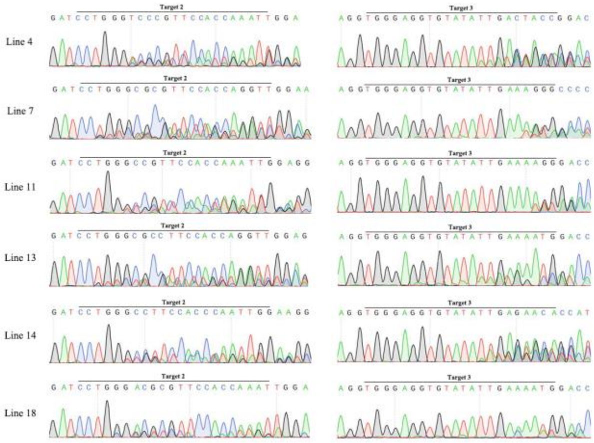

Figure S7. Sequence peaks of the target sites in pear.

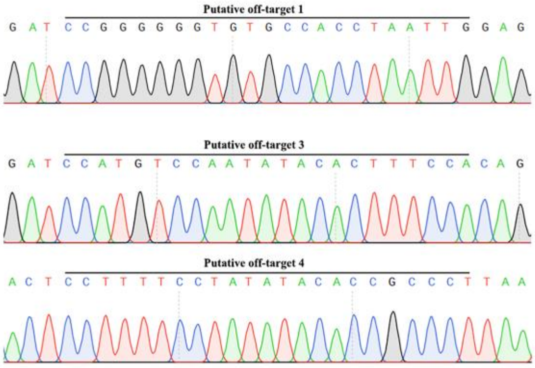

Figure S8. Sequence peaks of the potential off-target sites in pear.

Table S1. Information regarding AtPAT14 and putative PbPAT14s.

Name	Accession Number	E-value	Sequence
AtPAT14	AT3G60800.1		MHRSGTTMAWNVFKFCTALRGLGSIMILLVLGVVGVTYYAVVLTNYGPALSQGGLDSLAALTILILFHFLLAMLLWSY
PbPAT14-1	Pbr029181.1	6.00E-112	MHRSGVAMAWNVFKFCTALRGLGSVMILLVLGVVGVTYYTVVLTNYGPALYDGGLDSLVAVAVLILFHCLLVMLLW
PbPAT14-2	Pbr041901.1	2.00E-89	MAWNAFKLCTALRALGYVMVLVVVAIVGLTYYAVVLVNYLPSILSGGVNFVIAFPLLILFHFLLVMLLWSYFNVVLTD
PbPAT14-3	Pbr029311.1	3.00E-19	MSSPGLMNAVVFTGVAVMCVFNYSASVFRDPGRVPSTYMPDVEDSGNPMHEIKRKGGDLRYCQKCSHYKPARAHH
PbPAT14-4	Pbr029300.1	3.00E-19	MKGNTLNQKRPAPSLILQTRSPTHQPPNPRILPAENDTQSPGDAVVLTPVPPPGPPPPPENIPDTMTRSLGFSLPVTVVV

PbPAT14-5	Pbr010700.2	5.00E-17	MKFERFLSIPILMVFLLIGVVCYITVFIFIDDWVGLKSSAGSLNALVFIFLASLCLFSFFGCVLTDPGHVPASYVPDVEDSA
PbPAT14-6	Pbr025932.1	2.00E-14	MSRKKNQVWDAPSDDTAAAAPPPPERERLYLVWQGNNKFLCGGRIVFGHDAASLFLTSFLIGCPALAFCTRMLVMM
PbPAT14-7	Pbr021305.1	2.00E-14	MSRKKNQVWDAPSDDTAAAAPPPPERERLYLVWQGNNKFLCGGRIVFGHDAASLFLTSFLIGCPALAFCIRMLVMMR
PbPAT14-8	Pbr018617.1	3.00E-14	MRNNDMVRTQQERMYGNALPHQHSDSDRRIIDAPGPSLRLYQVWKGNNKFFFNGRIILGPDSRSLILTVSLIVVPVILF
PbPAT14-9	Pbr019497.1	5.00E-14	MTHLHNGQTPAKFILGGRLIFGPDARSLLVTLLLIIAPVIIFCVFVAWHLRHEFPSYNAGYAILVVAIVFTIYVLVLLFLTS
PbPAT14-10	Pbr027121.1	8.00E-14	MNAEPNHHLRHSPAGGAAAGGPELVRTYKTWKGSNIFFLGGRLIFGPDVRSLPLTVSLLTVPVAVFCIFVGRKLIDHLG

Table S2. Sequence of primers used for gene cloning and qRT-PCR.

Primer	Sequence (5'-3')
attB1	GGGGACAAGTTTGTACAAAAAAGCAGGCT
attB2	GGGGACCACTTTGTACAAGAAAGCTGGGT
14-1F	AAAAAGCAGGCTCCATGCATAGATCTGGAGTAGCCATG
14-1R	AGAAAGCTGGGTCTTAGAACTCTTGGGAATCAAACTCC
14-2F	AAAAAGCAGGCTCCATGGCGTGGAACGCGTTCAAG
14-2R	AGAAAGCTGGGTCTTATAAAGGCTGAAGTGCATTC
GAPcF	CACTTGAAGGGTGGTGCCAAG
GAPcR	CCTGTTGTCGCCAACGAAGTC
LBb1(BP)	GCGTGGACCGCTTGCTGCAACT
LP	TTCGACTATTCACCGTTCGAC
RP	TCCAATTAGGAGGCACAACAC
Action-F	TTGGTATGGGTCAGAAGG
Action-R	CTGTGAGCAGAACTGGGTG
qPbNCED3F	CTACAAGACACCGCCACCTT
qPbNCED3R	AGTGGGAGTTGAAGGTTGTTGA
qPbABI1F	AACTCAGCAGGTGCAGTGG
qPbABI1R	CCCAACGGTTTCTGGGGC
qPbMYB2F	ACTTGCCCGGTAGAACAGAC
qPbMYB2R	CTGGTACAGCTTCGGGCAA
qPbMYC2F	AATCTGTGGACGGACGACAA
qPbMYC2R	GGGTAGTCAGAAGTGGAGGC
qPbRD22F	GTTGGCAAAGGAGGAGTGTCC
qPbRD22R	TTTCCGTGGCTGCATACTTG
qPbSnRK2.2F	CCCTGCTGATTTGATGGACGA
qPbSnRK2.2R	TGCTGGTATAGTAGCCTCCTCA
qPbSnRK2.6F	AACTATGCGGTAGTGGAGCG
qPbSnRK2.6R	TGCTTTGGATCCACCTTCCC

Table S3. Sequence of primers used for CRISPR/Cas9 vector construction and mutant detection.

Primer	Sequence (5'-3')

gRT#T1	GCGCGCCCTCGGCTACGTGAgttttagagctagaaat
AtU3b#T1	TCACGTAGCCGAGGGCGCGCTgaccaatgttgctcc
gRT#T2	CAATTTGGTGGAACGCGCCCgttttagagctagaaat
AtU6-1#T2	GGGCGCGTTCCACCAAATTGCaatcactacttcgtct
gRT#T3	TGGGAGGTGTATATTGAAAAgttttagagctagaaat
AtU6-29#T3	TTTTCAATATACACCTCCCACaatctcttagtcgact
VecF	TACGCTGATCTCTTCCTTGCTGCT
VecR	TGTCCGACTTACCCCTGTTCTTGT
F1	TTCCGTCGGATCCCAAATGGGGA
R1	CAGAAAATGGAACAAGATCAAGA
F2	GGAATTTTAATTCGAGGTTTTA
R2	AAACAGAACAATGATGGCAAC
F3	AGTGATCCAGCAAATCAAAAAATAC
R3	GTGACAAGAGTTGTCTCAAGAAATG
OT1F	CTCGCAGTAAATTTGTAAAATCTGC
OT1R	CCAACACAATTAACAACCCATACAC
OT2F	CCAGGGAAGTGATCCTTGAATG
OT2R	TTAATTTGTGCCAGTCCGAATG
OT3F	AATGAAGTGTCGGTGGTTAGCT
OT3R	CCTTAAATTGCAGGTGGTGGAG
OT4F	CAAATGTCGTCGTACACTGCTT
OT4R	TAATTCTAGGCGTCTTGGGATG

Table S4. Potential off-target analysis at the three target sites of *PbPAT14* in pear.

Target	Off-target sites	Putative off-target sequences*	Putative off-target loci (*Pyrus betulifolia*)	Putative off-target loci (*Pyrus bretschneideri*)	Number of mismatched bases	Number of examined lines	Number of lines with mutations
**2**	1	**CCG**GGGGGTGTGCCACCTAATTG	Chr7: +11900769	Scaffold937.0: +148896	4	6	0
	2	**CCC**GGGCCCGTTCCACCACCGTG	-	Scaffold803.0: -132074	4	6	0
**3**	3	**CCA**TGTCCAATATACACTTTCCA	Chr16: - 323527	Chr16: - 4114455	4	6	0
	4	**CCT**TTTCCTATATACACCGCCCT	Chr3: -30953462	Chr3: -1285207	4	6	0

* Mismatched bases are shown in underline.

Table S5. Identification of *Arabidopsis* homologous genes involved in the ABA pathway of pear (*Pyrus bretschneideri* Rehd.).

Gene in Arabidopsis	Gene model in TAIR	Homologous genes in pear	Accession Number	E-value	Protein similarity (%)
AtNCED3	AT3G14440.1	PbNCED3	Pbr025271.1	0	68.06
AtABI1	AT4G26080.1	PbABI1	Pbr026157.1	2.00E-141	59.83
AtMYB2	AT2G47190.1	PbMYB2	Pbr008630.1	7.00E-86	53.65%
AtMYC2	AT1G32640.1	PbMYC2	Pbr042466.1	0	53.26

AtSnRK2.2	AT3G50500.2	PbSnRK2.2	Pbr007881.1	0	74.87
AtSnRK2.6	AT4G33950.1	PbSnRK2.6	Pbr040276.1	0	73.96

Table S6. Statistical information describing the mutant types of *PbPAT14* in the first generation.

Target	Plant ID	No. of clones sequenced	No. of clones with mutant alleles	No. of different mutant alleles
Target 1	4	8	0	0
	7	5	0	0
	11	8	0	0
	13	9	0	0
	14	7	0	0
	18	7	0	0
	Total	44	0	0
Target 2	4	8	6	3
	7	10	10	4
	11	9	9	3
	13	9	9	3
	14	8	3	2
	18	7	7	3
	Total	51	44	9
Target 3	4	8	8	3
	7	6	6	2
	11	6	5	2
	13	7	1	1
	14	10	10	4
	18	10	2	1
	Total	47	32	6
